# Operational Implementation of Remote Patient Monitoring Within a Large Ambulatory Health System: Multimethod Qualitative Case Study

**DOI:** 10.2196/45166

**Published:** 2023-07-27

**Authors:** Katharine Lawrence, Nina Singh, Zoe Jonassen, Lisa L Groom, Veronica Alfaro Arias, Soumik Mandal, Antoinette Schoenthaler, Devin Mann, Oded Nov, Graham Dove

**Affiliations:** 1 Department of Population Health New York University Grossman School of Medicine New York, NY United States; 2 Medical Center Information Technology NYU Langone Health New York, NY United States; 3 Rory Meyers College of Nursing New York University New York, NY United States; 4 Tandon School of Engineering New York University New York, NY United States

**Keywords:** digital health, remote patient monitoring, RPM, human-centered design, human-computer interaction, implementation science

## Abstract

**Background:**

Remote patient monitoring (RPM) technologies can support patients living with chronic conditions through self-monitoring of physiological measures and enhance clinicians’ diagnostic and treatment decisions. However, to date, large-scale pragmatic RPM implementation within health systems has been limited, and understanding of the impacts of RPM technologies on clinical workflows and care experience is lacking.

**Objective:**

In this study, we evaluate the early implementation of operational RPM initiatives for chronic disease management within the ambulatory network of an academic medical center in New York City, focusing on the experiences of “early adopter” clinicians and patients.

**Methods:**

Using a multimethod qualitative approach, we conducted (1) interviews with 13 clinicians across 9 specialties considered as early adopters and supporters of RPM and (2) speculative design sessions exploring the future of RPM in clinical care with 21 patients and patient representatives, to better understand experiences, preferences, and expectations of pragmatic RPM use for health care delivery.

**Results:**

We identified themes relevant to RPM implementation within the following areas: (1) data collection and practices, including impacts of taking real-world measures and issues of data sharing, security, and privacy; (2) proactive and preventive care, including proactive and preventive monitoring, and proactive interventions and support; and (3) health disparities and equity, including tailored and flexible care and implicit bias. We also identified evidence for mitigation and support to address challenges in each of these areas.

**Conclusions:**

This study highlights the unique contexts, perceptions, and challenges regarding the deployment of RPM in clinical practice, including its potential implications for clinical workflows and work experiences. Based on these findings, we offer implementation and design recommendations for health systems interested in deploying RPM-enabled health care.

## Introduction

Networked medical devices offer the potential for people affected by a variety of chronic conditions to monitor symptoms and physiological measures at home, and for the clinicians who treat them to gain more fine-grained and nuanced insight into their lived experience beyond visits to the clinic. Typically, this form of mobile health care (mHealth) is referred to as remote patient monitoring (RPM), defined as “the use of a non-invasive, wearable device that automatically transmits data to a web portal or mobile app for patient self-monitoring and health provider assessment and clinical decision-making” [1]. RPM proponents highlight opportunities for improved patient outcomes, decreased costs, and increased physician satisfaction [2-4]. It is also suggested that RPM will improve the timeliness of care, increase treatment adherence, and support personalized preventive medicine [5-7]. Recently, 2 key drivers have provided a strong motivation for health care practitioners in the United States to adopt RPM options as part of a growth in remote “virtual-first” health care offerings: the approval in 2018 for RPM to be reimbursed through the Centers for Medicare and Medicaid Services, providing the financial support of the largest US health care payer [8], and the rapid shift to remote provision of health care experienced during the COVID-19 pandemic [9-11].

Despite the growing enthusiasm for remote-supported clinical care delivery, to date, large-scale RPM implementation within health systems has been limited. Most studies on the use of RPM technology focus on smaller one-off initiatives—such as grant-funded research studies and disease- or department-specific pilot projects—and much of the evaluation focuses on proving clinical effectiveness in controlled settings and identifying issues with study quality (eg, the inability to conduct double blind trials, and study heterogeneity) [1,12]. As with digital health technology in general, the pragmatic use of RPM in clinical practice has been limited by issues of usability and acceptability, appropriateness for real-world disease management, integration into clinical and technical workflows, and cost-effectiveness [13]. Additionally, few best practices or practical guidelines exist to support the real-world implementation of RPM, or to set a foundation for successful use of this technology at-scale.

In response to this identified gap, health care services researchers have called for better understanding of the health care technology paradigm, including the need to design “person-centered” models that incorporate the needs and experiences of various stakeholders and are built with scalability and sustainability in mind. In this study, we explore the early implementation of a pragmatic operational RPM initiative across the ambulatory network of an academic medical center in New York City, focusing on the experiences, perceptions, and needs of patients and clinicians, with the goal of identifying key “person-centered” themes that can inform implementation recommendations for health systems interested in effectively deploying this technology.

## Methods

### Study Design

Our research used a multimethod qualitative approach consisting of semistructured interviews and design thinking workshops among participants (clinical staff, patients, and project implementation team members) of an ambulatory RPM initiative. These activities were conducted as part of a health information technology operational initiative to systematically expand the use of RPM technology to support blood pressure management for hypertensive patients within the expanded ambulatory network of one of the largest academic medical centers in the northeast United States.

### Population and Setting

The New York University Langone Health (NYULH) system is a large urban academic institution and tertiary care center, with a network of more than 15,000 clinicians in over 400 locations across New York, New Jersey, and Florida. The ambulatory networks consist of academic practices, community-based practices, and federally qualified health centers (FQHCs), as well as ambulatory surgery and rehabilitation centers. NYULH serves an ethnically and socially diverse population with a broad payor mix.

As part of the health system’s overall patient digital experience efforts, NYULH has invested in operational support to facilitate pragmatic implementation of RPM across its practices. NYULH’s electronic health record (EHR), Epic, supports both a “native” RPM integration—allowing data from Bluetooth-connected devices to “stream” through smartphones (eg, Android/GoogleFit or Apple/Healthkit) to the EHR—as well as manual data upload via the MyChart patient portal to support patients without smartphone access. MyChart also serves as a patient-facing tool to integrate RPM education and task management. RPM initiatives within the NYULH system are supported by the Medical Center Information Technology (MCIT) department, which specializes in the analysis, development, and delivery of applications and enterprise information technology solutions and includes personnel with expertise in software development (back-end and front-end programming), solution architecture, design, quality assurance, infrastructure engineering, and product operations and support (including Epic integration).

### Data Collection

#### Semistructured Interviews With RPM “Early Adopter” Clinicians

In 1962, communication theorist Everett Rogers introduced the concept of innovation “early adopters,” referring to the percentage of individuals who are quick to adopt a new technology, product, or idea [14]; these individuals offer unique perspectives and are considered integral to an innovation’s larger success. In health care, understanding the experiences of early innovation adopters can help facilitate the translation of these tools into diverse environments and support effective, safe, and sustained use.

In this study, early adopters of RPM technology were clinicians in the metro New York practice network (Manhattan, Brooklyn, and western Long Island) who used RPM between 2018 and 2021, prior to the health system’s system-wide RPM initiative. This included maternal-fetal medicine (blood pressure monitoring), pediatric endocrinology (continuous glucose monitoring), lung transplant (temperature, spirometry, pulse oximetry, and weight), bariatric surgery (weight, blood pressure, and glucometer), and primary care FQHCs (blood pressure), each of which independently piloted monitoring initiatives as part of grant-related quality or clinical effectiveness initiatives with the support of the MCIT team. Clinicians at practices with early experience using RPM technologies in disease management were invited to participate in a 30-minute semistructured interview as part of an RPM operational quality improvement effort to identify early resource needs and potentially successful strategies to inform scaling RPM more broadly within the ambulatory health system (see Multimedia Appendix 1). Interview prompts were guided by literature review and focused on clinician experiences, workflows (clinical and technical), and barriers and facilitators. Prompts were reviewed with content experts, the RPM implementation team, and ambulatory clinical leaders. The interviews were conducted in December 2020 and May 2021. Interviews were audio recorded and detailed notes were also taken.

#### Speculative Design Workshops

Speculative design and futuring workshops have emerged as popular research practices in human-centered and human-computer interaction design. Building on critical design practices and design fictions [15,16], these workshops aim to spark discussion and encourage reflection on the potential implications of emerging technologies. Workshop activities provide a creative environment in which participants can safely explore potentially challenging topics, without a commitment to developing practical solutions to their real-world problems. Outputs may include visual or textual narratives, or prototypes that illustrate alternate futures.

For this research, four 90-minute speculative design workshops were developed to study the following patient identities: (1) early adopters of health technology; (2) patients with chronic diseases (eg, diabetes); (3) parents; and (4) other caregivers. Patients aged between 30 and 76 years were recruited from New York metropolitan area and New Jersey. The workshops were adapted from the speculative design approach in More&More Unlimited’s Investing in Futures framework, which focuses on envisioning future-facing case scenarios and experiences using RPM technology (see Multimedia Appendix 2) [17]. Each session began with participants sharing care experiences, specifically positive encounters with health care providers and moments when they felt cared for. The second activity was world building, here we asked participants to wonder together about what a good future might look like using cards with prompts. The prompts were divided into 8 categories: wearables, health management, smart homes, community, data storage, communication, cost, and health care system. The third and last activity was to build a day in the life of 1 person in the new future using their assigned patient identity. Sessions were conducted from March to August 2021. Transcripts from the workshops were used to identify themes and collect quotes from participating patients.

### Data Analysis

This study followed the Standards for Reporting Qualitative Research reporting guideline for qualitative studies [18]. All data sources were recorded (audio logged and transcribed) and deidentified prior to analysis. For qualitative analysis, we applied a hybrid inductive-deductive approach described by Fereday and Muir-Cochrane [19]. First, the data were analyzed using an inductive coding process to identify and iteratively develop and refine emergent themes and codes. Subsequently, a deductive approach focusing on barriers and facilitators was applied to elucidate particular themes relevant to challenges in early RPM implementation. A representative subset of data (2 interviews and 1 speculative design session) was independently coded by 3 primary coders (LLG, ZJ, and NS). Codes were iteratively discussed with the larger research group to (1) review major and minor themes and points of divergence and convergence, (2) establish and refine the code book, and (3) determine thematic saturation. Data were then independently recoded by the primary coders. An additional coder (GD) independently read the coded data for accuracy and to identify cross-cutting themes.

### Ethical Considerations

This study was conducted as part of a quality improvement and patient safety evaluation in conjunction with the NYULH MCIT Department. Researchers completed an NYU Langone Health Institutional Review Board–approved quality improvement self-certification.

## Results

### Overview

Twenty-four stakeholders participated in the study across data collection methodologies. Thirteen early adopter clinicians (n=11 physicians, n=1 nurse practitioner, n=1 registered nurse; n=7, 53% identified as female; average 16 years in clinical practice) represented pediatric endocrinology, maternal-fetal medicine, weight management clinics, pulmonary transplant, internal medicine, and federally qualified health centers. Twenty-one patient representatives participated in 4 speculative design sessions (n=5 early adopters, n=5 parents, n=5 caregivers, and n=6 managing a chronic condition; n=11, 53% identified as female).

We present our findings across three main themes: (1) data collection and practices; (2) proactive and preventive care; and (3) health disparities and equity. We also identify evidence for mitigation and support to address challenges in these areas (Textbox 1 and Multimedia Appendix 3).

**Themes and subthemes identified.**

**Data collection and practice**

Clinical impacts of real-world measures
Issues of data sharing, security, and privacy

**Proactive and preventive care**

Proactive and preventive monitoring
Proactive interventions and support

**Health disparities and equity**

Tailored and flexible care
Implicit bias

**Mitigation and support for remote patient monitoring-enabled health care**

### Data Collection and Practice

#### Overview

The first finding centered on experiences and concerns regarding sharing data about personal health measures that are generated in settings other than the clinic. Two areas in particular were highlighted among participants: (1) clinical impacts of real-world measures and (2) issues of data sharing, security, and privacy.

#### Clinical Impacts of Real-World Measures

The potential for RPM to expand clinical diagnosis and management capabilities through the collection of more ecologically valid measures in a wider range of real-world contexts that better reflect a patient’s lived experience were noted by both clinicians and patients.

My mom gets really anxious around doctors, and being in a doctor’s office, and so it always looks like her blood pressure is through the roof when she’s there. She has to manually track her blood pressure at other times so she can go to the doctor and be like, “No I’m not. This isn’t my standard. I have a very normal blood pressure. I’m just a little freaked out by you trying to give me blood pressure medication.”

Speculative design session 1, participant #2, female

A physician noted similar data quality worries, pointing in particular to concerns about older patients being prescribed multiple medications for hypertension and the challenge of using clinical blood pressure values to accurately gauge the overall effects of the medication:

Allowing a patient to log at home, particularly for older patients... Some patients read high in clinic and are fine at home.

Clinician #6, male

At the same time, participants also noted concerns regarding the feasibility of using remote monitoring technologies, and the burdens placed on patients to routinely collect these data, particularly around access, language, and digital and health literacies.

Tech access and literacy is a global concern.... [Patients] may not be able to use RPM technologies to accurately self-measure blood pressure or glucose.

Clinician #6, male

This impacted the clinician’s confidence in using the patient-reported data to make clinical decisions. Additionally, attending to RPM health data can have unique psychological impacts on patients, both positive and negative; on one hand, patients responded positively to data within “normal” values, as it provided a reassurance that everything is going well, however, when the data suggested something outside of expectations it was seen as a source of worry for patients:

I think it is a little bit of some mental warfare for [patients], because if their number is a little low and they can’t get the number they know, then they’re obviously worried that there’s some problem...they’re worried about [transplant] rejection.

Clinician #2, female

The physician noted being unsure of the best way to counsel patients on the impacts of this “mental warfare,” or how to adjust home monitoring to optimize patient well-being.

#### Issues of Data Sharing, Security, and Privacy

A subtheme of particular importance to patient participants was that of the relationship between RPM and data sharing, security, and privacy. For many patient participants, RPM data represented an opportunity for better connected health care experience. In particular, there was interest in the capacity to share data between primary care physicians and medical specialists:

You know how when you get older you see one specialist after another? Specialists are starting to be able to coordinate MyChart [the patient portal] and all these other things. But a lot of doctors, well you know, I uploaded it to this one and that one, I ran through permission but then by the time you get to the doctor’s office and it’s like “I can’t see your records.”

Speculative design session 3, participant #3, female

For other patients, there is an apparent trade-off between the benefits of data connectedness and the risks of privacy breaches:

I know that there are significant privacy issues with these [digital health tools], but I feel like it’s an area, especially with all of these wearable devices and everything, it just makes sense to begin to connect more, to be able to pull it together and get a better level of care as a result.

Speculative design session 4, participant #2, male

For others, data collection and sharing raised significant questions about privacy and security, in particular regarding how data might be used by or shared with other companies:

I just feel like companies that will be collecting all this information, let’s say in a future scenario monetary system, what if they’re selling your data to third parties? That would really kind of be a concern.

Speculative design session 2, participant #4, female

As a result of these impressions, many patients expressed reservations about using RPM technologies regularly in their care. Conversely, clinicians did not routinely mention data privacy or security as an issue in their data management or clinical practices, focusing instead on the aforementioned challenges of data quality and interpretability in clinical contexts.

### Proactive and Preventive Care

#### Overview

A specific area of reflection from both patients and providers revolved around the impact of RPM on the practice of providing medical care, and the potential shift from reactive to proactive care provision enabled by the technology. Subthemes on this topic include: (1) proactive and preventive monitoring and (2) proactive interventions.

#### Proactive and Preventive Monitoring

Clinicians discussed a number of potential opportunities that RPM data might facilitate with regard to population health and preventive monitoring of their patients, such as the ability to programmatically identify patients that are struggling to maintain a suggested program of treatment or who are not being adequately served. In this way, they would hope to reduce the likelihood that these patients would end up in the emergency room (clinician #6, male). Similarly, the potential for clinicians to proactively communicate with their patients in response to data generated by RPM-enabled health care was highlighted as a key benefit by multiple providers. One physician pointed to the way that blood pressure data come directly as a message to their EHR in basket, allowing them to respond using the patient portal (clinician #1, female). When a series of readings were considered beyond normal values, clinicians commented they could reach out immediately to the patient and ask them to schedule a visit more promptly than they would have otherwise (clinician #7, male). At the same time, clinicians also drew attention to concerns that patients who do not actively submit their remote health data might receive less proactive attention from clinicians, and potentially be excluded from outreach initiatives that relied on these data to identify patients. For example, if a patient fails to submit remote blood pressure data in a particular week, they will not receive a call from the nurse, as in this workflow they would not be identified in the EHR (clinician #7, male). Clinicians also noted the unintended consequences of the increase in patient-generated messaging around their RPM data: “It can take several hours to go through everything [in the EHR]” (clinician #1, female).

From the patient perspective, a key challenge highlighted during the workshops was the potential negative effect of this proactive communication:

Maybe somebody doesn’t want the doctor calling them every time there’s a little spike.... They’ll be like, everything’s fine, leave me alone, I’ll call you if there’s a problem. I can kind of see that maybe being a little invasive.

Speculative design session 2, participant #4, female

There was broad concern among participants in the patient workshops that an increased reliance on data and technology to monitor progress might lead to a reduction in personal care and remove opportunities for empathetic connections: “My biggest concern with this would be that if doctors are getting all these numbers all the time, it doesn’t dehumanize you” (speculative design session 1, participant #4, male):

You know, once we start adding more machines and more technology, people lose that personal sense of connection and that’s just something I’m not willing to sacrifice.

Speculative design session 2, participant #3, female

#### Proactive Interventions and Support

Building on the opportunities offered by proactive communication, providers also discussed how RPM can support proactive interventions, specifically between scheduled visits:

[Before RPM] if patients were just getting started on medications the [staff] would bring them back next week to review logs.... Now they don’t have to do that, they can still see them in two weeks, and can [review remotely] in between visits.

Clinician #2, female

For 1 clinician, RPM promised opportunities to specifically support health behavior education:

I’m hoping it helps in the sense that we’re really able to get our patients involved in their care, and it’s not just every three months I get my sugar checked when I come to the clinic. If they’re doing it on a daily basis then it helps them to realize it’s important and the education helps them understand that everything they do impacts their health. I’m hoping that it helps our patients to understand that everything they do really does make a difference.... I’m hoping it helps us track and make patients aware of their choices.

Clinician #4, female

Patients were keen to highlight how data from RPM might also help in their own decision-making to support healthier choices and behavior:

And so the more data that I have I feel like I can make better decisions, right? Something that I was thinking about doing was making a food journal, because I really don’t know how many calories I’m actually taking in. And so, is there a better way to do that?

Speculative design session 1, participant #3, male

Another patient postulated that RPM data might provide the basis for an insurance incentive to encourage people to follow through with suggestions made during their annual check-up (speculative design session 1, participant #5, male).

While proactive interventions were generally considered a positive opportunity, providers did highlight challenges in this new workflow, including billing and reimbursement. This was particularly relevant among the FQHC providers:

FQHCs are a little different, we can’t bill directly for RPM but we want to use the technology.... I’m concerned about sustainability. If we can’t bill for it, how does it pay for itself?

Clinician #5, male

Experiences in other practices led clinicians to comment on the tendency of patients to disregard RPM data, for example by switching data streams off rather than positively responding and adopting healthier behaviors, thereby limiting its potential effectiveness (clinician #1, female). Overall, clinicians expressed concerns about being asked to provide more care as a result of RPM, “We’re asking them to do more work between visits when they’re not compensated, and that’s hard” (clinician #5, male).

### Health Disparities and Equity

#### Overview

Both clinicians and patients discussed the role of RPM technologies in addressing or exacerbating inequalities in health care, with the main areas of interest and concern regarding (1) tailored and flexible care and (2) implicit bias.

#### Tailored and Flexible Care

For clinicians, RPM presents an opportunity to directly address health inequities through expanded access to care and more effective, tailored health care usage. One clinician discussed the potential of targeting care delivery to specific underserved women of color who were disproportionately affected by gestational diabetes and could benefit from tailored monitoring (clinician #2, female). The opportunity to replace arduous visits to the clinic with a telehealth virtual visit was considered an incentive for patients to present to and stay in care:

For patients [living] in other boroughs it’s so hard to come into Manhattan. Parking. If their family member takes them. It’s a lot. So, we will sometimes do video visits for people in this area who just [can’t] come in.

clinician #8, female

In the context of RPM specifically:

We kind of, for better or worse, use [RPM] a little bit like a reward system.... If you’re not doing your monitoring you have to come in, because we can’t do a proper visit with you.

clinician #8, female

However, this potential of RPM to address health disparities was viewed as potentially limited for a number of reasons, including barriers related to the technology and its appropriateness or easy use for diverse patients. A lack of culturally and contextually congruent technology, and wrap around services for RPM-enabled health care, was considered a major barrier to its ability to be effectively tailored to diverse patients who may otherwise have benefited:

My main concern is that it’s not all in Spanish. The majority of our patients speak Spanish.

Clinician #4, female

Other participants reflected that, in order to support flexible and tailored care for diverse patients, patients and clinicians need to overcome a range of barriers already associated with health technologies. For example, many of the RPM devices that integrate most effectively with the hospital’s EHR system are only available through more expensive and comprehensive insurance plans, and so may be unavailable to patients that could benefit the most (clinician #3, female). Similarly, 1 physician described how current billing mechanisms for RPM-enabled health care often result in out of pocket fees, which make it an inaccessible option for those with limited income (clinician #1, female).

#### Implicit Bias

A particular area of challenge in RPM-enabled health care practice is centered around identifying patients who might be considered good candidates for RPM. Clinicians noted that they thought certain patients were probably better suited for RPM programs than others, often based on individual assessments of digital literacy skills, health literacy, language abilities, and proactive participation in care. This was highlighted by a physician who said that patients would most likely be selected for RPM programs based on the question, “Do I think they can do it?” explaining that this would be a soft assessment that takes into consideration financial concerns as well as digital literacy and health literacy (clinician #7, male). For another physician, patients considered a potentially bad fit for RPM would be “people who don’t want to use technology” or who faced “language barriers” (clinician #2, female). A third clinician reported:

Tech-savvy persons and high-literacy people are more likely to use [RPM]...[we are] less likely to offer RPM to people who are less likely to use it.

Clinician #4, female

This process of patient identification and selection for inclusion in RPM programs was not explicitly identified to be a form of systematic bias by either clinicians or patients; rather, it was most often discussed as a factor that contributed to the patient’s activity or success within the RPM programs.

### Mitigation and Support for RPM-Enabled Health Care

Participants identified possible approaches to supporting opportunities and mitigating challenges posed by novel RPM practices. These typically related to new roles and responsibilities that might be created to better support interactions with technology systems and health care administration. For example, 1 patient highlighted how cultural competence and concordance can be important to equity in health care delivery:

I have over the past few years intentionally sought out doctors who were people of color, particularly Black Americans and doctors who were women. I just find that in doing so, I think my health care in general is usually better. There’s questions and discussions and cultural sensitivities that I find are being addressed in general when I have doctors of color and doctors who are women.

Speculative design session 4, participant #5, female

Clinicians noted that having a range of clinical staff who speak a patient’s first language may be critical to RPM, as it demands a higher frequency of communication and support (clinician #4, female).

Both clinical providers and patients highlighted a variety of roles or services that could be provided to support patient navigation, advocacy, and competence. One patient stated:

I feel like one of the things about the health care system right now is that it is so confusing to read about your benefits or your insurance and what’s covered by what and how you qualify for things.

Speculative design session 1, participant #2, female

Clinicians cited community health workers (CHWs) as helpful to connecting with vulnerable populations, in part because they may already be making home visits with these patients (clinician #4, female). In particular, CHWs were considered potential digital advocates who could act as intermediaries with RPM technology:

CHWs [could perform] teaching around what to expect and how to use the [devices], assessing access to wireless communications...to help them troubleshoot.

Clinician #6, male

However, it was noted that additional training would be required for this staff, as these tasks were considered outside of the current scope of their work:

CHWs are great to work with. They can help facilitate the MyChart sign up and encourage them to use [RPM] in a way that is helpful. It would definitely require some training in how to use it, though.

Clinician #5, male

## Discussion

### Summary of Results

In this study, we present the challenges and considerations associated with the transition of a health care system to a care delivery model enabled by RPM technology. Using data triangulated from an institution currently undergoing the pragmatic deployment and scaling of RPM in practice, we identified 3 main themes of interest and concern to RPM stakeholders: (1) novel data collection practices and concerns; (2) proactive and preventive care models; and (3) health disparities and health equity. Our work also identified opportunities for mitigation and support for RPM-enabled care, particularly around new work roles and resources to support those who are engaging in this type of care delivery. This study contributes to the existing literature on remote monitoring by capturing the experiences and perspectives of various key stakeholders (including clinicians, patients, caregivers, other health staff) in a health care system that is actively undergoing a care delivery transition enabled by RPM technology.

### Implications for the Pragmatic Deployment of RPM Within Health Systems

This work highlights a few key areas of consideration for health care systems or practices that are considering undertaking RPM-enabled care transformations. The first is around ensuring a “successful” remote monitoring experience for patients and clinicians. Substantial research in the areas of digital health technology points to the clear need for an improved overall user experience of these technologies, from back-end data integration and interoperability to front-end product design [20-22]. As endorsed by both patients and clinicians in this study, the ideal RPM experience is driven by the efficient transmission of validated, trustworthy data in an environment that is user-friendly, humanistic, and information secure. At the same time, priorities of different stakeholders regarding these technologies may not overlap, and in some instances may be in direct conflict. Our study showed that, while clinicians and patients discussed similar themes regarding RPM, their individual concerns or perspectives were often conflicting—while clinicians endorsed wanting to have more ability to access data for patient care, patients were concerned about their clinicians monitoring them continuously; and while clinicians expressed concerns about being overly contacted by their patients between visits, patients themselves were worried about losing their personal relationships with their doctors. Clearly identifying and, where possible, aligning the diverse needs and preferences of RPM stakeholders can facilitate a more acceptable, usable RPM program; it can also reduce potential areas of friction that might contribute to nonadoption, abandonment, or unintended negative effects on patient experience or patient-clinician relationships.

The second area highlights the novel practice structures that can be implemented to better support RPM-enabled care; specifically, the opportunity for new work roles around digital health navigation and advocacy, and the shifted nature of the patient-clinician relationship. In our study, both patients and clinicians identified the potential that digital advocates (including CHWs) might have on improving the experience of care using RPM. Our findings reflect other work indicating how the introduction of data-intensive technology such as RPM can bring with it important changes to clinicians’ work, including increasing administrative labor and shifting temporal work patterns (eg, work outside work or “pajama time”) [23-25]. New practices that empower members of the health care team beyond physicians, or that automate the more routine aspects of these interactions while allowing physicians to engage in specialized practice, will be needed to successfully implement data- or monitoring-intensive technologies [26]. Examples that have been suggested elsewhere include the use of AI-enabled chatbots to help perform clinical analyses and provide an initial response to data that are within expected ranges, and the implementation of big data analytics and machine learning more widely within health care information technology [27]. However, it should also be noted that participants in the patient workshops strongly indicated how important a close relationship with their clinical provider was for them; patients would prefer to securely share data with the clinicians they know and trust and make collaborative decisions with empathetic physicians. Similarly, while clinicians are keen to encourage patients’ engagement in their own care, they too indicated how acting on RPM data should be collaborative. These findings echo others in the digital health literature as well as the larger human-computer interaction research field, which warns of the danger of focusing too strongly on data and highlights the positive impacts of more emotional and experiential self-reporting to health and well-being [28-31]. This suggests that, at its core, technology-centered care such as RPM should include an element of humanistic, mutually beneficial comanagement, which should include not only the patient-provider-technology triad, but the expanded team of digital care advocates (both trained and lay) as well.

The third area of critical consideration is that of “inclusive RPM.” Integrating health equity considerations into interventions and prevention programs has been identified by the US Centers for Disease Control and Prevention as a key factor in improving public health [32]. While digital health technologies such as RPM have the potential to improve health equity, our findings also reflect concerns that the uneven application of these technologies in clinical contexts may exacerbate existing health disparities or potentially create new sources of digitally-mediated inequities [33]. Our findings also indicate that the promise RPM technologies offer toward directly addressing health inequities by expanding access and tailoring health care may be more fragile outside the constraints of research studies. Our findings highlight clinicians’ expectations for RPM technologies to help facilitate a more flexible and tailored approach to health care provision, which includes virtual appointments and interventions, and which reduces the impacts of travel and unpaid leave that disproportionately impact patients from underserved communities. However, we also identified a number of barriers to implementing RPM-enabled care in a way that effectively addresses disparities. These barriers are consistent with existing literature showing that digital health care interventions are generally more accessible to socioeconomically advantaged groups, and that health technology programs often neglect digitally-mediated factors at the community or society level which influence health disparities [34,35].

### Limitations

This study has several limitations. It reflects the experience of a particular health care institution at a given moment in the pragmatic development of its remote monitoring program. Participants were identified through pragmatic convenience sampling methodologies and may not reflect the overall composition of either the institution itself or the larger pool of patients and providers engaging with RPM. Data were collected through a variety of methods and may reflect a number of biases, including interviewer bias and response bias. Due to small sample sizes and risks of participant identification, we are unable to provide more detailed information on specific clinic- or department-level experiences that may have differed between practices. Strengths of the study are that it reflects the experiences of key stakeholders (patients and clinical providers) participating in the real-world implementation of a digital health intervention, using an organic multisource qualitative approach to ensure a diversity of stakeholders, approaches, and contexts were captured.

### Conclusions

In this paper, we present an inquiry into the challenges and considerations associated with the transition of RPM-enabled health care from research studies into clinical practice. Our analysis of qualitative data from patients, clinicians, and health staff identified 3 main themes related to the pragmatic implementation of this technology, including issues around data collection and review practices, proactive and preventive care experiences, and technology-mediated health disparities and inequity. We further identified opportunities for mitigation and support of the challenges and opportunities raised, including building skills, capacity, and diversity among the future clinical workforce engaged in RPM-related care. Ultimately, the introduction of RPM-enabled health care poses particular design and implementation challenges for current practices, creating a potentially unbalanced patient-provider-technology triad that can disrupt practice patterns and norms and affect the experience of care for both patients and clinicians. Understanding and responding to these challenges can help improve its acceptability, scaled use, and sustainability in health care delivery.

## Appendix

Multimedia Appendix 1 Semistructured interview guide.

Multimedia Appendix 2 Speculative design toolkit.

Multimedia Appendix 3 Themes, subthemes, and representative quotes.

## Abbreviations

CHW: community health worker
EHR: electronic health record
FQHC: federally qualified health center
MCIT: Medical Center Information Technology
NYULH: New York University Langone Health
RPM: remote patient monitoring
