# Supplementary material for: Operational Implementation of Remote Patient Monitoring Within a Large Ambulatory Health System: Multimethod Qualitative Case Study
Source: JMIR Hum Factors. 2023 Jul 27;10:e45166. doi: 10.2196/45166 (PMC10415949; doi:10.2196/45166)
Supplement: Multimedia Appendix 1 [file humanfactors_v10i1e45166_app1.docx]

*Semistructured interview guide*

Remote patient monitoring early adopter baseline interview

Interviewer: We are interested in learning more about FHC clinicians’ perspectives, opinions, and needs for the successful use of remote patient monitoring (RPM) in their clinical practice.  Remote patient monitoring is defined as “technologies to enable monitoring of patients and their medical conditions *outside of conventional clinical setting*s, such as in the home or in a remote area”.

As this technology becomes more common in healthcare delivery, we are exploring ways in which our practices can successfully integrate and implement these tools in their patient care and clinical workflows.

There are no right or wrong answers, and your responses in this interview are confidential.

- How do you currently use RPM with your patients?
  - what types of devices do you use?
  - do you use any “unconnected” devices (ie: home BP cuffs without internet connectivity, manual glucose or BP logs, other tools)
- How do you envision you might use RPM in your clinic?
  - In general, what kinds of clinical care are/would be best suited for remote patient monitoring?
- How do you decide who can/should be a candidate for RPM?
  - What patients are considered “good” candidates?
  - Why might you/might you not prescribe RPM for a patient?
- What have patients shared with you about RPM?
  - How have their experiences been?
  - What do you think are barriers for patients? Facilitators?
  - What support do you think patients need to successfully use RPM?
- In general, what support do you think clinicians need to effectively use RPM in their clinics?
  - What are some of the barriers to effective use in your practice currently?
- In what ways are your staff (nurses, MAs, front of house) currently involved in RPM care/work?
  - In what ways could they be involved? What services would they provide?
- How do you think RPM might alleviate and/or exacerbate disparities in healthcare?
  - How might that be addressed?


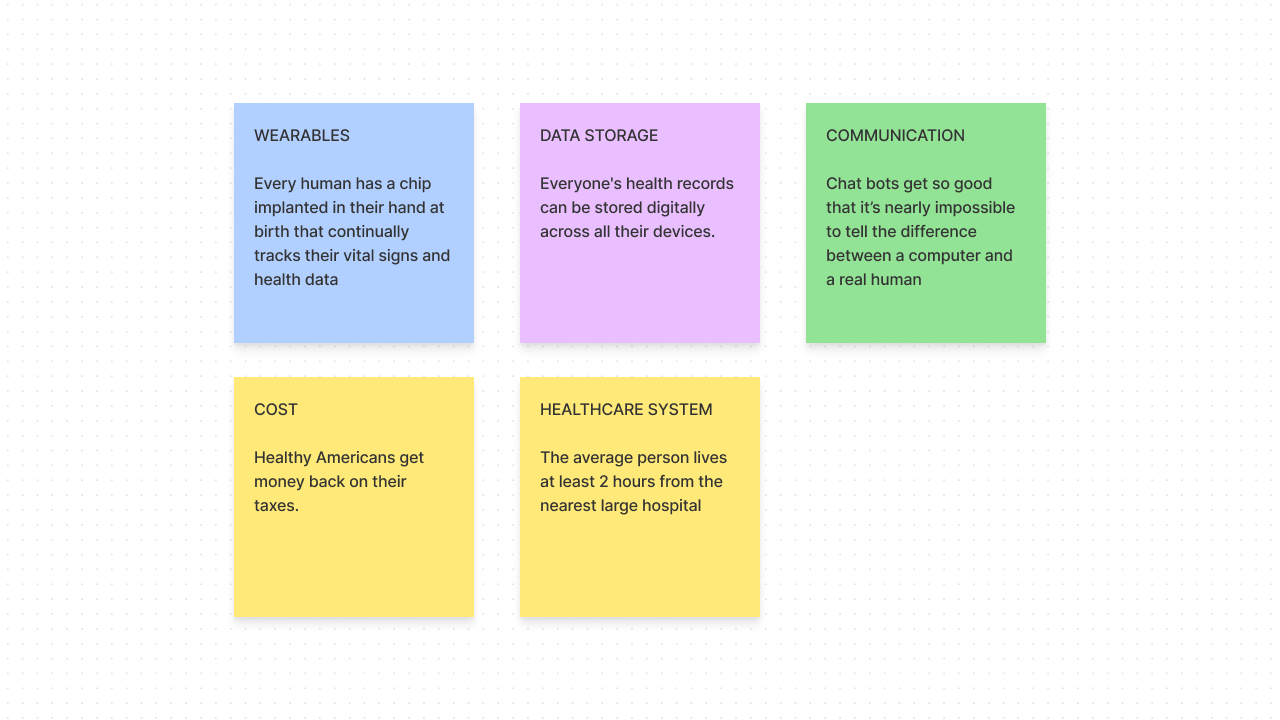


Group 2: Parents


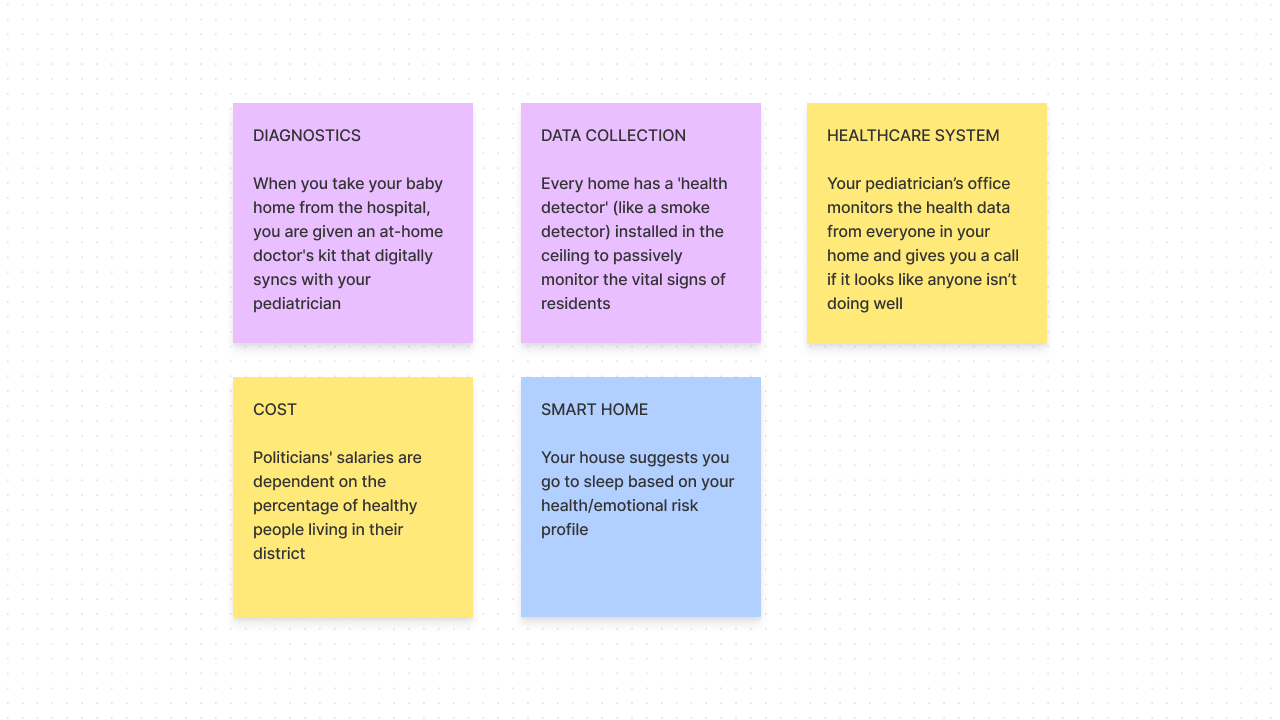


Group 3: Caregivers *(nonprofessional, of someone in their own family)*


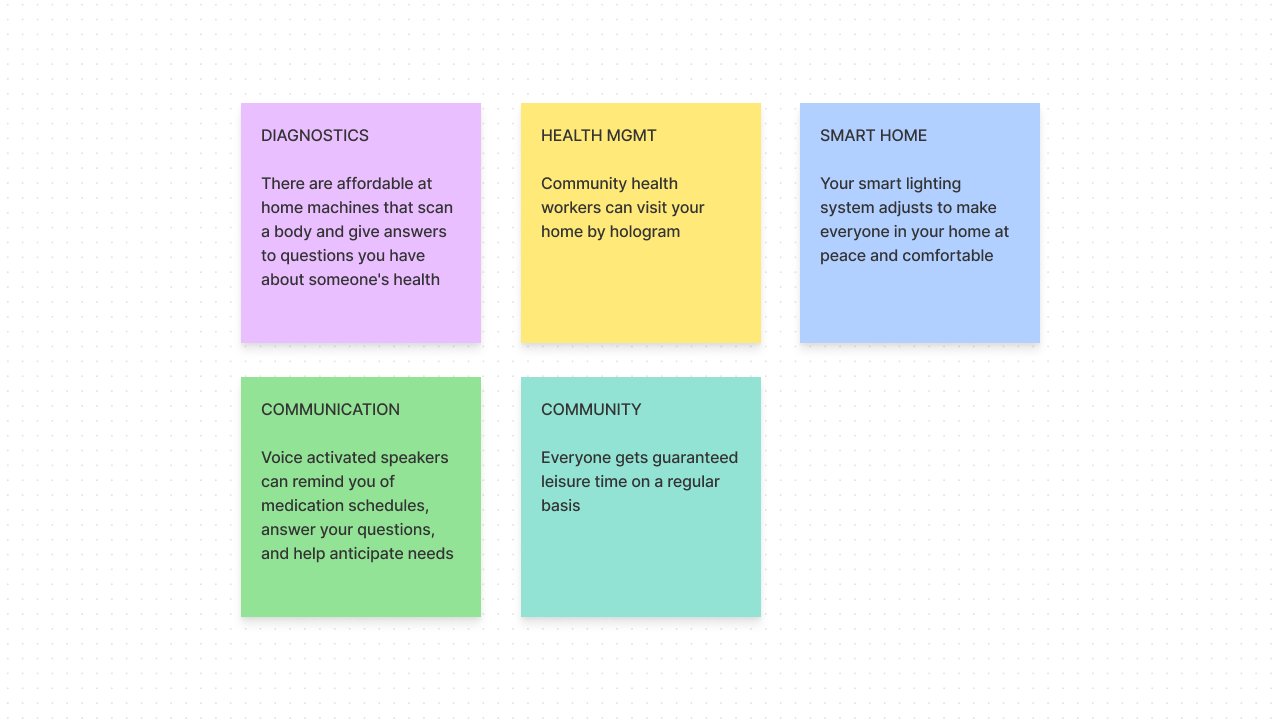


Group 4: Managing a Chronic Condition


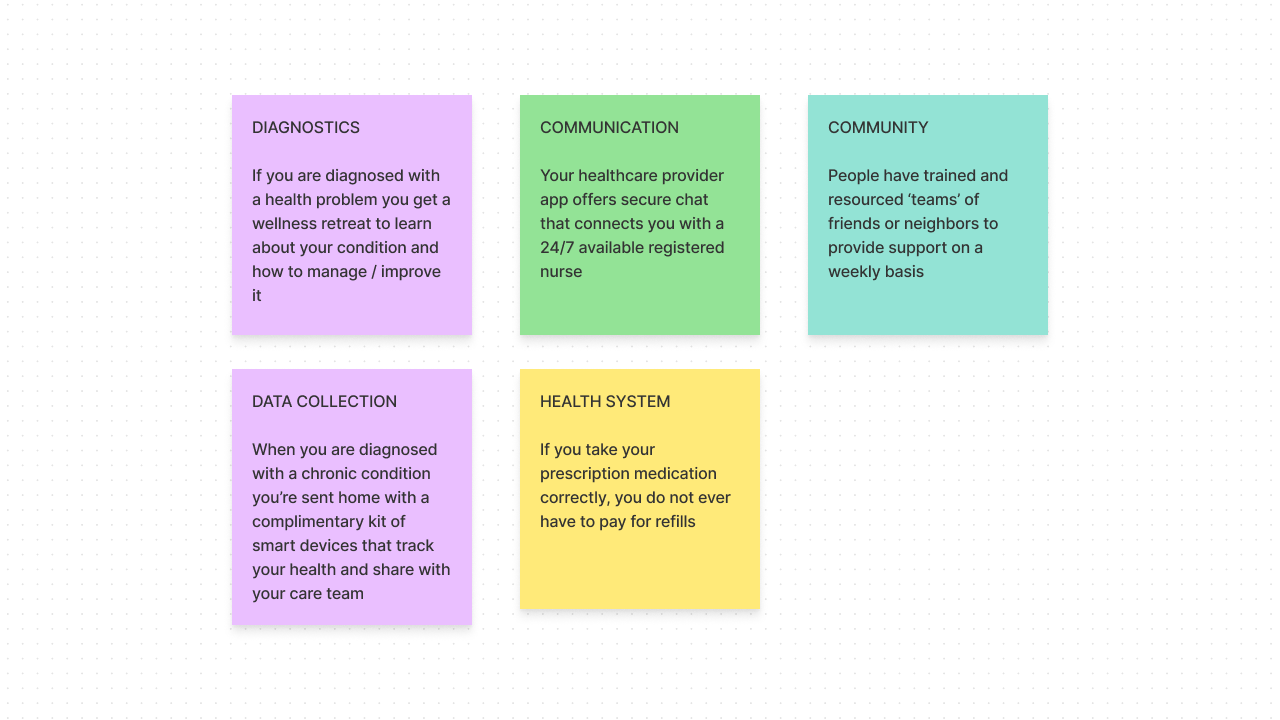


[PROMPTS AS NEEDED]

- Feel free to debate the meaning of your cards and re-imagine them as you wish.
- What impact would these cards have on people, community and culture?
- What about the healthcare system? What does your relationship with your clinician look like? How do you get care?
- What kinds of technology are necessary to enable this future? How do people get access to this tech?
- What about emotional wellbeing, religion, spirituality and wellness?
