# Supplementary material for: Operational Implementation of Remote Patient Monitoring Within a Large Ambulatory Health System: Multimethod Qualitative Case Study
Source: JMIR Hum Factors. 2023 Jul 27;10:e45166. doi: 10.2196/45166 (PMC10415949; doi:10.2196/45166)
Supplement: Multimedia Appendix 2 [file humanfactors_v10i1e45166_app2.docx]

*Speculative design toolkit*

# Research Guide

## Welcome [5 mins]

Thanks for joining us today for this session.

I’m [NAME], a researcher for a healthcare system. Your participation here doesn’t have any connection to care you get at any medical institution.

- We will be together for about 90 minutes.
- We will start by talking about our experiences with healthcare, then will do a working session to envision a better future and will close with a few questions.
- We will take a few short stretch breaks, but if you need a bio break at any time feel free to mute yourself and go off camera.
- We have some silent observers joining us, and a special guest illustrator!
- We are recording this conversation for notetaking purposes, but anything you share will be kept confidential. We will not attribute your comments to you.

Care Experiences [20 mins]

Let’s talk for a few minutes about times we felt cared for.

1. Tell us about one positive experience you had with a healthcare provider. (doctor, nurse practitioner, lab technician, office staff, etc.)
2. Now share about a time you felt cared for (in general). This can be a moment from any context in your life.
3. Do you notice anything interesting in our stories? What stands out to you?

## World Building [20 mins]

Now we are going to transition to the creative part, where we will wonder together about what a good future might look like.

Here is how this is going to work: as thought starters, I will share some ‘cards’ that include characteristics of the future we will build together. You can channel your inner science fiction writer and image what this world would look like – but let’s stick to a good future we would actually want to live in.

Jacques is going to join us for this portion of the conversation to illustrate the world as we build it.

Ready?

1. Go ahead and discuss the items on the screen and talk together about what you want your world to look like.

Group 1: Early Health Tech Adopters
