# Supplementary material for: Operational Implementation of Remote Patient Monitoring Within a Large Ambulatory Health System: Multimethod Qualitative Case Study
Source: JMIR Hum Factors. 2023 Jul 27;10:e45166. doi: 10.2196/45166 (PMC10415949; doi:10.2196/45166)
Supplement: Multimedia Appendix 3 [file humanfactors_v10i1e45166_app3.docx]

*Themes, subthemes, and representative quotes*

| Themes | Subthemes | Quotes |
| --- | --- | --- |
| Data collection and practice | Clinical impacts of real-world measures | "My mom gets really anxious around doctors, and being in a doctor's office, and so it always looks like her blood pressure is through the roof when she's there. She has to manually track her blood pressure at other times so she can go to the doctor and be like, "No I'm not. This isn't my standard. I have a very normal blood presure. I'm just a little freaked out by you trying to give me blood pressure medication" [speculative design session 1, participant #2, female] |
|  |  | “Allowing a patient to log at home, particularly for older patients...Some patients read high in clinic and are fine at home” [clinician #6, male] |
|  |  | “Tech access and literacy is global concern...  [Patients] may not be able to use RPM technologies to accurately self-measure blood pressure or glucose,” [clinician #6, male] |
|  |  | “I think it is a little bit of some mental warfare for [patients], because if their number is a little low and they can’t get the number they know, then they’re obviously worried that there’s some problem… they’re worried about [transplant] rejection [clinician #2, female] |
|  | Issues of data sharing, security, and privacy | “You know how when you get older you see one specialist after another? Specialists are starting to be able to coordinate MyChart [the patient portal] and all these other things. But a lot of doctors, well you know, I uploaded it to this one and that one, I ran through permission but then the time you get to the doctor’s office and it’s like ‘I can’t see your records’” [speculative design session 3, participant #3, female] |
|  |  | “I know that there are significant privacy issues with these [digital health tools], but I feel like it’s an area, especially with all of these wearable devices and everything, it just makes sense to begin to connect more, to be able to pull it together and get a better level of care as a result” [speculative design session 4, participant #2, male] |
|  |  | “I just feel like companies that will be collecting all this information, let’s say in a future scenario monetary system, what if they’re selling your data to third parties? That would really kind of be a concern.” [speculative design session 2, participant #4, female] |
| Proactive and preventive care | Proactive and preventive monitoring | “It can take several hours to go through everything [in the EHR]” [clinician #1, female] |
|  |  | “Maybe somebody doesn’t want the doctor calling them every time there’s a little spike…They’ll be like, everything’s fine, leave me alone, I’ll call you if there’s a problem. I can kind of see that maybe being a little invasive” [speculative design session 2, participant #4, female] |
|  |  | “My biggest concern with this would be that if doctors are getting all these numbers all the time, it doesn’t dehumanize you” [speculative design session 1, participant #4, male] |
|  |  | “You know, once we start adding more machines and more technology, people lose that personal sense of connection and that’s just something I’m not willing to sacrifice”. [speculative design session 2, participant #3, female] |
|  | Proactive interventions and support | “[Before RPM] if patients were just start on medications the [staff] would bring them back next week to review logs….  Now they don’t have to do that, they can still see them in two weeks, and can [review remotely] in between visits.” [clinician #2, female] |
|  |  | “I’m hoping it helps in the sense that we’re really able to get our patients involved in their care, and it’s not just every three months I get my sugar checked when I come to the clinic. If they’re doing it on a daily basis then it helps them to realize it’s important and the education helps them understand that everything they do impacts their health. I’m hoping that it helps our patients to understand that everything they do really does make a difference...I’m hoping it helps us track and make patients aware of their choices” [clinician #4, female] |
|  |  | “And so the more data that I have I feel like I can make better decisions, right? Something that I was thinking about doing was making a food journal, because I really don’t know how many calories, I’m actually taking in. And so, is there a better way to do that?” [speculative design session 1, participant #3, male] |
|  |  | “FQHCs are a little different, we can’t bill directly for RPM but we want to use the technology…I’m concerned about sustainability. If we can’t bill for it, how does it pay for itself?” [clinician #5, male] |
|  |  | “We’re asking them to more work between visits when they’re not compensated, and that’s hard” [clinician #5, male] |
| Health disparities and equity | Tailored and flexible care | “For patients who live in other boroughs it’s so hard to come into Manhattan. Parking. If their family member takes them. It’s a lot. So, we will sometimes do video visits for people in this area who just [can’t] come in” [clinician #8, female] |
|  |  | “We kind of, for better or worse, use [RPM] a little bit like a reward system…If you’re not doing your monitoring you have to come in, because we can’t do a proper visit with you” [clinician #8, female] |
|  |  | “My main concern is that it’s not all in Spanish. The majority of our patients speak Spanish” [clinician #4, female] |
|  | Implicit bias | Patients would most likely be selected for RPM programs based on, “Do I think they can do it”, explaining that this would be a soft assessment that takes into consideration financial concerns as well as digital literacy and health literacy [clinician #7, male]. |
|  |  | Patients considered a potentially “bad” fit for RPM would be “people who don’t want to use technology” or who faced “language barriers” [clinician #2, female]. |
|  |  | “tech-savvy persons and high-literacy people are more likely to use [RPM]...[we are] less likely to offer RPM to people who are less likely to use it” [clinician #4, female] |
| Mitigation and support for RPM-enabled healthcare |  | “I have over the past few years intentionally sought out doctors who were people of color, particularly Black Americans and doctors who were women. I just find that in doing so, I think my health care in general is usually better. There’s questions and discussions and cultural sensitivities that I find are being addressed in general when I have doctors of color and doctors who are women” [speculative design session 4, participant #5, female] |
|  |  | “I feel like one of the things about the health care system right now is that it is so confusing to read about your benefits or your insurance and what’s covered by what and how you qualify for things” [speculative design session 1, participant #2, female] |
|  |  | “CHWs [could perform] teaching around what to expect and how to use the [devices], assessing access to wireless communications…to help them troubleshoot” [clinician #6, male] |
|  |  | “CHWs are great to work with. They can help facilitate the MyChart sign up and encourage them to use [RPM] in a way that is helpful. It would definitely require some training in how to use it, though” [clinician #5, male] |
